# The phylogenetic position of ctenophores and the origin(s) of nervous systems

**DOI:** 10.1186/2041-9139-6-1

**Published:** 2015-01-13

**Authors:** Gáspár Jékely, Jordi Paps, Claus Nielsen

**Affiliations:** Max Planck Institute for Developmental Biology, Spemannstrasse 35, 72076 Tübingen, Germany; Department of Zoology, University of Oxford, South Parks Rd, Oxford, OX13PS UK; The Natural History Museum of Denmark, University of Copenhagen, Universitetsparken 15, DK-2100 Copenhagen, Denmark

**Keywords:** Ctenophore, Nervous system evolution, Cnidarian, Sponge, *Trichoplax*, Neuropeptide, Metazoan phylogeny, DEG/ENaC channels, MicroRNA, Ciliary photoreceptor

## Abstract

Ctenophores have traditionally been treated as eumetazoans, but some recent whole genome studies have revived the idea that they are, rather, the sister group to all other metazoans. This deep branching position implies either that nervous systems have evolved twice, in Ctenophora and in Eumetazoa, or that an ancestral metazoan nervous system has been lost in sponges and placozoans. We caution, however, that phylogenetic-tree construction artifacts may have placed ctenophores too deep in the metazoan tree. We discuss nervous system origins under these alternative phylogenies and in light of comparative data of ctenophore and eumetazoan nervous systems. We argue that characters like neuropeptide signaling, ciliary photoreceptors, gap junctions and presynaptic molecules are consistent with a shared ancestry of nervous systems. However, if ctenophores are the sister group to all other metazoans, this ancestral nervous system was likely very simple. Further studies are needed to resolve the deep phylogeny of metazoans and to have a better understanding of the early steps of nervous system evolution.

## Introduction

**Figure 1:**
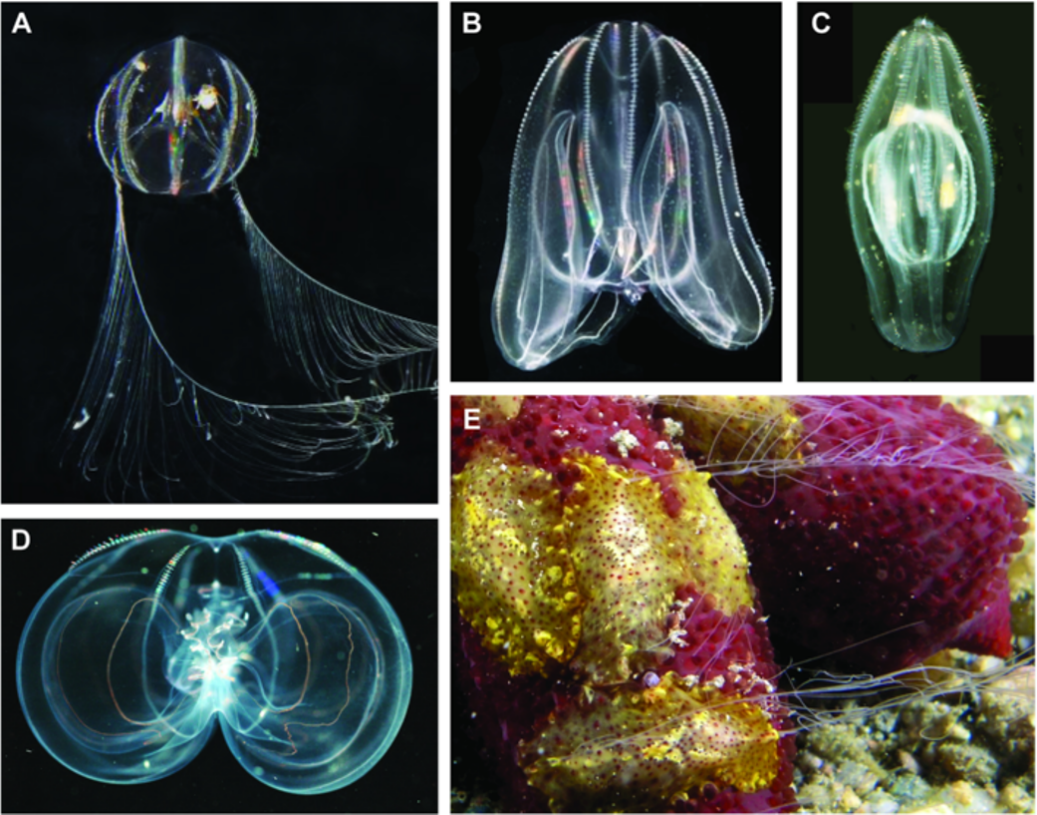
**Ctenophore diversity. A**, *Pleurobrachia bachei* (D Kent); **B**, *Mnemiopsis leidyi* (K Brandt); **C**, *Beroe gracilis* with *Pleurobrachia pileus* in the stomach (C Marneff); **D**, *Thalassocalyce inconstans* (L Madin); **E**, *Coeloplana astericola* (yellow-brown with thin, branched tentacles) on the red sea-star *Echinaster luzonicus* (D Fugitt). Reproduced with permission from D Kent, K Brandt, C Marneff, L Madin and D Fugitt.

Ctenophores (also known as comb jellies or sea gooseberries) are free-living marine organisms. They represent a non-bilaterian lineage of Metazoa (besides cnidarians, sponges, and placozoans) of particular importance for understanding early animal evolution. Most ctenophores look like ghostly, transparent jellies with eight comb rows of iridescent, compound cilia used in swimming, and many have long, retractile tentacles with a comb row of side branches (Figure 1). The side branches of the tentacles are covered with colloblasts; cells which contain vesicles with a sticky substance used in capturing prey organisms, such as copepods. The rather familiar ‘sea gooseberries’ (*Pleurobrachia*) probably represent an ancestral type, which is pelagic and shows almost the same morphology in both the adult and in the newly hatched cydippid stage. All described ctenophore species hatch as a cydippid stage (except the highly specialized *Beroe*, which lacks tentacles in all stages) but adults of the different species show enormous variation in form. One line of morphological specialization in adult ctenophores involves enlargement of the oral lobes and gradual loss of the tentacles, as seen in *Mnemiopsis.* A number of open-ocean species have very large oral lips and only small comb rows and are extremely fragile. *Beroe* lacks tentacles and feeds on other ctenophores or medusae, which may be swallowed whole. *Beroe* also has the remarkable ability to ‘bite’ into jellies with their macrociliary ‘teeth’. A specialized group has a flattened benthic adult stage that crawls on the ciliated extended lips of the wide mouth and extends the tentacles through small funnels formed by lateral folds of the mouth; this group lacks the hallmark comb rows. Some of these species are brightly colored and are found living on other animals, such as corals or sea stars. Newly hatched juveniles produce sperm and a number of small eggs, which develop into normal juveniles. After a period of growth, the small individuals become sexually mature again and produce numerous larger eggs.

Unlike sponges, ctenophores have a gut with digestive enzymes lined with an epithelium, a complex nervous system and a complicated system of muscles [1]. The ctenophore nervous system is organized into an epithelial and a mesogleal nerve net and two parallel nerve cords in the tentacles [1]. They have sophisticated sensory cells, including putative photo-, mechano- and gravi-receptors [2, 3]. The nervous system controls the activity of cilia, bioluminescent flashes and muscular contractions [3, 4]. Ctenophores use ciliary comb rows for locomotion and the beating frequency and the arrests of the cilia are controlled by dedicated neuronal systems [3]. The cydippid *Euplokamis* has giant axons that run longitudinally along the eight comb rows and control fast backward and forward escape responses [5].

**Figure 2:**
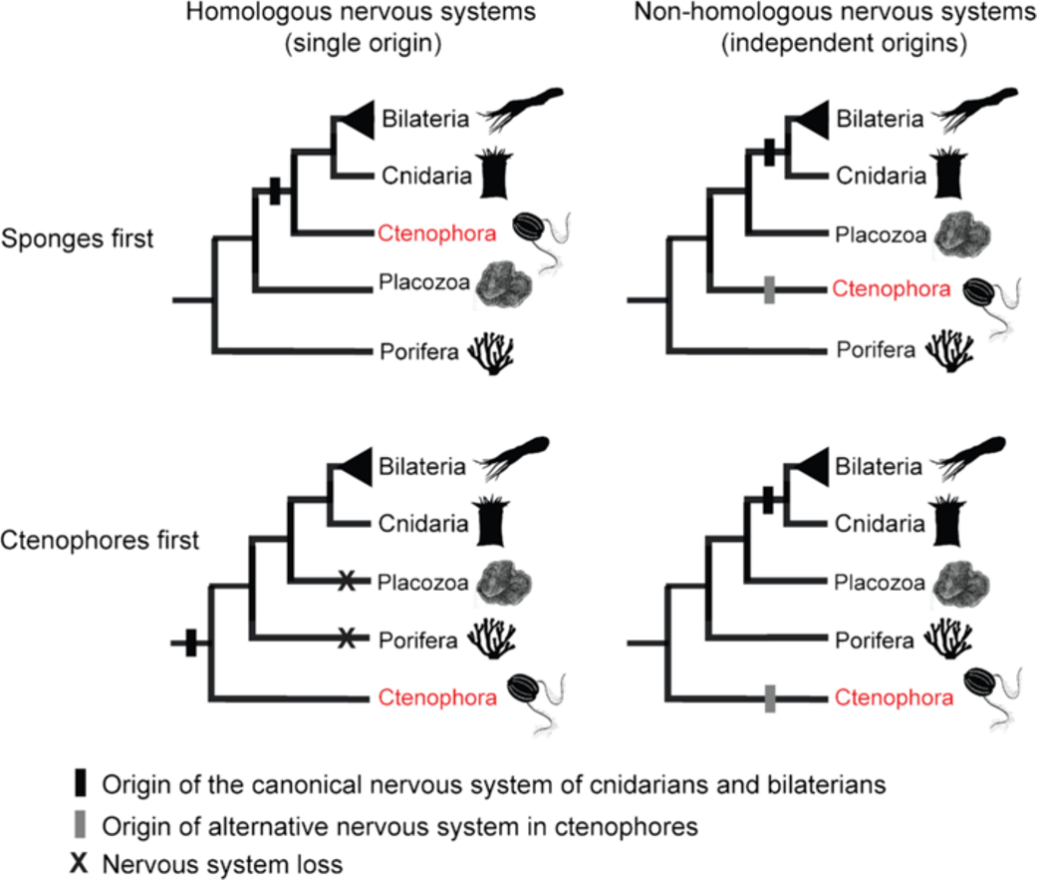
**Four scenarios for the origins of nervous systems.** Four scenarios for the origins of nervous systems in the Animal Kingdom depending on the homology of their components and the phylogenetic position of ctenophores. If nervous systems are homologous across metazoans, and if ctenophores are the earliest-diverging animals, then nervous systems were lost in sponges and placozoans. In contrast, if nervous systems are not homologous across animals then they arose more than once, a result that is not made more or less likely by any of the possible placements for ctenophores. Vignettes from phylopic.org and C Nielsen.

Ctenophores have been classified as eumetazoans, often as the sister group of the cnidarians, but also as the sister group of the bilaterians. Two recent whole-genome analyses of the ctenophores *Mnemiopsis leidyi* [6] and *Pleurobrachia bachei* [7] have indicated, however, that the ctenophores do not belong to the Eumetazoa but are instead the sister group to all other metazoans, a position more typically occupied by the sponges. The placement of ctenophores as a sister to all other metazoans was supported by earlier phylogenetic studies [8–12], while other work indicated sponges as the sister lineage to all other metazoans [13–16]. The recent analyses of ctenophore genomes also support the non-canonical phylogeny based on the absence from ctenophores of key eumetazoan characters, such as *Hox* genes and microRNAs. If this phylogeny is correct, then nerves and muscles must either have evolved independently in Ctenophora and Eumetazoa (for simplicity, referring to Cnidaria plus Bilateria throughout the paper), or these systems evolved in the metazoan ancestor and have been lost in sponges and placozoans, lineages without any trace of synaptically connected nerve cells. Alternatively, ctenophores may be a sister group to cnidarians or to bilaterians or to eumetazoans, and their placement outside the eumetazoans may be due to artifacts affecting phylogenetic reconstruction (Figure 2).

The phylogenetic position of Placozoa is also unstable. Some previous studies using mitochondrial markers [17, 18] pointed to an early split of *Trichoplax* within a monophyletic Diploblastica clade (Porifera, Placozoa, Ctenophora and Cnidaria). However, recent larger datasets dismissed the monophyly of diploblasts and have placed placozoans in different branches, although never as first-splitting metazoan. These positions include *Trichoplax* as sister to cnidarians [6, 7, 12, 19], sister to bilaterians [14], or sister to cnidarians, ctenophores and bilaterians [16, 20].

Here we highlight potential technical problems related to the placement of ctenophores in the metazoan tree and discuss scenarios of nervous system evolution under different phylogenetic frameworks. We also discuss how studying sponges and placozoans can contribute to our understanding of nervous system evolution.

## Review

### The uncertain phylogenetic position of Ctenophora

While several phylogenomic analyses [6, 7, 11, 12] support the position of ctenophores as the sister lineage to all other animals, other works considered this branching contentious in light of methodological issues. One of the main concerns is long-branch attraction (LBA), a well-known artifact in phylogenetic tree reconstruction that incorrectly places fast-evolving branches close to the root of the tree. Other factors often associated with phylogenetic errors are the use of too divergent out-groups, high levels of missing data (‘gaps’ due to incomplete sequencing), contamination and poor taxon sampling. Some of these methodological issues have previously been highlighted as potentially affecting the placement of ctenophores [13–16, 20], and each of these issues needs to be tackled with different strategies.

To alleviate these problems, both genome papers used different-sized alignments with different species sampling, and a variety of analytical methods [6, 7]. The use of distant out-groups has been shown to increase the LBA effects in different studies, and in the specific case of metazoans they reduce the supports for the early splitting branches on the animal phylogeny [13, 15, 16]. Both genomic studies on ctenophores compare the effect of using out-groups with different taxonomic compositions. Not surprisingly, different phylogenomic analyses produce contradicting results. In both studies, when more metazoan taxa are included using expressed sequence tag (EST) data, most of the analyses show reduced support for the ‘ctenophores first’ topology; in the case of Ryan *et al*. [6], the Bayesian analyses of the EST dataset removing distant out-groups supports the topology of ‘sponges first’. Some of the Bayesian analyses performed by Ryan *et al*. [6] did not complete (converge) even after months of computer run time, hence their results must be considered unreliable. Remarkably, some of the authors performed further analyses despite their huge computational cost [21]. In these, the exclusion of problematic taxa from the original EST matrix resulted in the analyses eventually converging; these trees suggested that sponges diverged first, if less distant out-groups were used. Overall, it seems that under conditions that deal better with methodological problems (a greater representation of ctenophore species, exclusion of problematic taxa and use of closer out-groups), the trees tend to support the ‘sponges first’ view of animal phylogeny, suggesting that the alternative solution is caused by a phylogenetic artifact.

The age of the last common ancestor (LCA) of the extant ctenophores may be partially responsible for the problems of placing them in the metazoan tree. The low variability of 18S in ctenophores suggests that their LCA was more modern than those of other animal phyla, perhaps as recent as the Cretaceous-Tertiary (K-T) boundary (66 Mya [22]). This modern LCA is indicated by a long branch leading from the other animals to the ctenophore LCA. Groups with such long stems are more difficult to place in phylogenies as there is no information regarding the appearance of homoplasy along this branch, causing what could be called a LCA-LBA effect. There is also little hope that wider ctenophore taxon sampling will break this long branch, as was the case for nematodes or acoels [23–25], since the range of ctenophores sampled in the two genome studies already captures the known diversity of the group.

The use of alternative phylogenetic markers (such as rare genomic changes [26]) can also be explored in order to escape the statistical traps of phylogenetic artifacts. Ryan *et al*. [6] presented a tree based on gene-content analysis. This tree supports the ‘ctenophores first’ topology, however, such analyses can be strongly distorted by extensive gene loss or divergent genomes [27]. Tellingly, the genome-content tree places *Ciona* artificially deep in the tree, as a sister to all deuterostomes rather than as sister group to the vertebrates. *Mnemiopsis* has undergone a similar magnitude of gene loss as *Ciona* (Figure S9 in Ryan *et al*. [6]), and this may also have affected its placement in the gene-content tree.

To use the absence of particular classes of genes, such as *Hox* genes and microRNAs, as phylogenetic evidence of ctenophores being the sister group to all other metazoans is also problematic, given that it is difficult to distinguish loss from primitive absence. *Hox* genes may have been lost from sponges [28], and the loss of microRNAs is not uncommon [29, 30] Both ctenophores and placozoans lack microRNAs and essential components of the microRNA processing machinery (*Drosha* and *Pasha* are absent from *Mnemiopsis*, *Pasha* is absent from *Trichoplax*). However, the full processing machinery and microRNAs are present in sponges [31, 32], indicating that the absence of the microRNA pathway is due to secondary loss, at least in placozoans. These data indicate that some of the ‘rare genomic changes’ may not be rare enough, and one should exercise extreme caution when using such characters for phylogenetic inference [29].

Overall, determining the precise branching order of non-bilaterian groups will have to overcome these confounding factors, such as LBA and the effects of out-group and in-group sampling. The existence of a hypothetic LCA-LBA effect should also be explored. More robust and rarer genomic changes (such as derived gene fusions or insertions) may also be identified upon further sampling and in-depth analyses. Further work is needed before we can feel comfortable with any single proposed arrangement of the early animal lineages.

### Comparisons of the ctenophore and eumetazoan nervous systems

The comparison of the molecular and cellular characteristics of the ctenophore and eumetazoan nervous systems can inform us about their common or independent origins. The two ctenophore genome papers provide a wealth of new sequence information to consider [6, 7].

Moroz *et al*. [7] make a strong case for the independent origin of the ctenophore nervous system. One observation they put forward in support of this idea is the apparent absence of neurotransmitter receptors and several classic neurotransmitters, such as serotonin and acetylcholine, and of neuronal marker genes from *Pleurobrachia*. We suggest that these observations need to be considered with some caution for two reasons. First, some of the genes are not expected to be present in ctenophores because they are known to be bilaterian-specific (*neurogenin* and *neuroD* [33]). Second, some genes and transmitters may be there in other ctenophores, but are absent from *Pleurobrachia*; at least some neurotransmitters have been shown to have a patchy distribution within the ctenophores, including acetylcholine and adrenaline. Pharmacological studies found evidence for a role of these transmitters in the control of luminescence in some ctenophores [34]. The presence of acetylcholinesterase in the *M. leidyi* genome also indicates that acetylcholine may be present in this species [6]. These observations suggest that the absence of at least some neuronal genes and neurotransmitters from ctenophores may be due to secondary loss rather than primitive absence.

It is to be noted, however, that the presence of neurotransmitter pathways and ‘neuronal’ genes does not necessarily indicate the presence of a nervous system, since both sponges and placozoans contain several such genes, including enzymes in the pathways for making serotonin, dopamine (sponges [35, 36]) or noradrenaline and adrenaline (*Trichoplax* [19, 37]). Sponges and ctenophores share the use of glutamate as a signaling molecule with other metazoans, and in sponges gamma-aminobutyric acid (GABA) receptors are present and work antagonistically to glutamate [38], which differs from *Pleurobrachia* [7]. Ionotropic glutamate receptor genes have also been identified in both sponges and placozoans [19, 36, 37], and their numbers have greatly expanded in ctenophores [7].

Ctenophores also contain several putative neuropeptide precursors [7] and large numbers of G-protein-coupled receptors (GPCRs), suggesting the existence of a complex peptidergic signaling repertoire. Neuropeptides have not yet been found in sponges, but are present in all other metazoans, including the placozoan *Trichoplax* [39, 40]. The reported ctenophore neuropeptide precursors show no homology to any known metazoan neuropeptide family, suggesting at first that they developed independently in ctenophores. However, the cross-phylum conservation of neuropeptides is often limited to a few residues, and cnidarians, placozoans and bilaterians have few recognizably orthologous peptides [39, 40]. The lineage-specific alteration of several neuropeptides beyond the limits for recognition of homology has also been observed in bilaterians (for example, flatworms [41]). The lack of similarity of ctenophore and other metazoan peptides may be due to the long period of time since those genomes diverged and the relatively high (but not extreme) rate of protein sequence evolution (comparable to *Drosophila*) in ctenophores (Tables S12 and S13 in Ryan *et al*. [6]). More detailed studies of ctenophore neuropeptides (including mass-spectrometric analyses and receptor identification) will be needed to understand their evolution.

Even if neuropeptides and their receptors are homologous, their presence is not sufficient evidence for nervous system homology since *Trichoplax*, an animal that lacks a morphologically recognizable nervous system, also possesses these molecules. What may be the function of neuropeptides in placozoans? A recent detailed morphological study of *Trichoplax* described neurosecretory gland cells containing neuropeptides but lacking classical synapses [42]. These gland cells also have a cilium and are putatively sensory, reminiscent of non-synaptic sensory-neurosecretory cells described in some eumetazoans [43]. The sensory stimulation of such cells is thought to lead to neuropeptide release that may affect surrounding cells in a paracrine fashion [42, 43]. Reconstructing the history of these cell types will further our understanding of the ancestral states of (pre)neural systems. Importantly, as the example of *Trichoplax* illustrates [42], the correct interpretation of comparative genomic data will also require sound morphological and functional studies.

The discovery of a family of putative peptide-gated ion channels in ctenophores also presents a very interesting avenue of future research. The *Pleurobrachia* genome encodes many DEG/ENaC channels (referred to in Moroz *et al*. [7] as ASICs, a name reserved for a chordate-specific subfamily of DEG/ENaC channels), a family of ion channels ancestrally gated by neuropeptides [44]. Although DEG/ENaC channels can have diverse gating mechanisms, including gating by protons, as in the ASICs, they can mediate fast peptidergic neurotransmission in cnidarians and some bilaterians [44–46]. If some of the ctenophore DEG/ENaC channels turn out to be peptide receptors, this would indicate the presence of fast peptidergic neurotransmission in the common ancestor of ctenophores and other metazoans. It will also be interesting to see whether placozoan DEG/ENaC channels are used for fast peptidergic transmission in this organism. If this is the case, then the presence of fast neurotransmission in an organism need not necessarily imply the presence of a morphologically recognizable, structurally complex nervous system.

One key feature of a nervous system that distinguishes it from an assembly of neurosecretory cells communicating in a paracrine fashion is the presence of electrical and chemical synapses that allow fast and direct communication between connected neurons. The ctenophore genome studies have revealed the presence of specific components of the pre-synapse, notably including five proteins that form the core of active zones in eumetazoan synapses, including the Rab3-RIM-Munc13 complex, ELKS and liprin-alpha proteins [47]. The presence of these molecules suggests that chemical synapses were already present in the last common ancestor of ctenophores and eumetazoans. However, Munc13 and liprin-alpha orthologs ([GenBank: XP_002110349] and [GenBank: XP_002108580], respectively) are also present in *Trichoplax*, an organism that supposedly lacks active zones, cautioning again that it is very difficult to pinpoint any specific component that defines a nervous system.

Additionally, the ctenophore nervous system expresses a large diversity of innexins [7], proteins that form gap junctions in most bilaterian invertebrates and cnidarians [48], but are absent from sponges and placozoans [49]. The high diversity and neuronal expression of innexins in ctenophores and other metazoans is suggestive of the presence of electrical synaptic communication between neurons in their last common ancestor. One has to caution, however, that gap junctions are also involved in the exchange of chemicals between cells [50] and have developmental roles [51], showing that their presence alone does not prove the presence of electrical synapses between neurons. Moreover, innexins and gap junctions can be lost completely, as from *Nematostella*, other anthozoans and scyphozoans [48], indicating that their presence or absence is not a reliable phylogenetic marker either.

Future studies at the level of neural cell types promise to reveal more about the structural complexity of the nervous system at deep nodes of the metazoan tree. Besides neurosecretory cells, ctenophore sensory systems may also show homologies to the eumetazoan nervous system at the level of neuron types. For example, unlike sponges and placozoans, but like all other groups of metazoans, ctenophores contain opsins [52]. The ctenophore apical organ harbors morphologically distinct ciliary photoreceptors [2], and the genome encodes components of the full ciliary phototransduction cascade [52], suggesting further similarities of ctenophores to cnidarians and bilaterians in their sensory physiology. A recent phylogenetic study showed that the ctenophore opsins group together with either the ciliary or the Go-coupled opsin family [53], members of which are commonly associated with ciliary photoreceptors [54, 55]. Ciliary photoreceptors may thus represent a sensory neuron type shared by ctenophore and eumetazoan nervous systems.

What do these similarities tell us about the origin of nervous systems? Overall, the detailed molecular and cellular similarities are compatible with the single origin of the ctenophore and eumetazoan nervous systems [47, 56]. However, given that there are very few shared characters that are nervous-system specific and are absent from sponges and placozoans, the independent origin of complex nervous systems from a simple precursor state in the common metazoan ancestor cannot be ruled out. Further comparative studies will be needed to assess the conservation, or lack thereof, of developmental mechanisms and the neuronal cell-type complexity in their common ancestor. For example, it would be interesting to test using knockout or knockdown approaches whether the neuronally expressed *SoxB* gene also has an essential role in nervous system development in ctenophores, as recently shown for the cnidarian *Nematostella vectensis* [57]. It would also be interesting to analyze the expression in ctenophores of conserved apical-organ-specific genes recently identified in *Nematostella* [58]. The detailed comparison of the rich repertoire of sensory cell types in ctenophores [3] and eumetazoan sensory neurons also represents an interesting subject for future research. The identification of sensory receptors (such as mechano- and thermo-sensory channels) in ctenophores and comparisons to other non-bilaterians and bilaterians may reveal other conserved sensory receptor classes, besides the opsins.

### The influence of phylogeny on origin scenarios

Although homology of complex biological traits can be assessed without knowing the exact phylogeny, based on statistical principles (well established for molecular sequences, more difficult for complex cellular and organismic data such as gene expression patterns), clarifying the deep branching order of metazoans will undoubtedly help to resolve the problems surrounding the origin of nervous systems. If ctenophores do turn out to belong to the eumetazoans, the homology of nervous systems will also gain phylogenetic support. If, however, ctenophores are sister to the rest of metazoans, this implies that sponges and placozoans have lost the nervous system, or that nervous systems evolved independently in ctenophores and eumetazoans. We now consider these two possibilities.

The likelihood of the loss of a nervous system has to be judged relative to the complexity of the starting condition. If the metazoan common ancestor had a complex, highly integrated nervous system with several sensory cell types and motor responses, we think that the loss of such a system twice independently (in sponges and placozoans) is highly improbable. There are only two examples of the complete loss of the nervous system in Metazoa, both in parasites. The extremely reduced parasitic myxozoan cnidarians lack any trace of a nervous system [59] and the rhizocephalan cirripede crustaceans also lack a nervous system in the adult stage. In this latter instance, however, the free-living larval stage has a normal crustacean central nervous system [60].

In the case of myxozoans and rhizocephalans, nervous system loss was due to an extreme life history evolution, for which there is no evidence in sponges and placozoans. Quite to the contrary, both sponges and placozoans have at least one life cycle stage with active ciliary locomotion and have developed systems for coordinating ciliary activity and body movements. The free-swimming ciliated larvae of several sponges perform helical phototaxis, using a specialized ring of pigment-covered light-sensory cells [61]. This active behavior is performed more efficiently using neuronal systems in several eumetazoan larvae, yet there is no trace of neuronal connections in the sponge larva [62, 63]. Furthermore, in the case where more rapid signaling occurs, for example in the glass sponges (Hexactinellida), a different system of epithelial conduction is used, involving syncytial tissues and propagated calcium potentials [64–66]. Studies on the sponge *Ephydatia muelleri* have shown its ability to sense and react to environmental stimuli [67]. Under the loss of nervous system scenario such systems for rapid signaling, sensing and reaction would have evolved *de novo*, following the loss of a preexisting system for rapid signaling. *Trichoplax* is also free-living, with active ciliary locomotion and a ventral ciliated surface that functions as a gut with extracellular digestion [68]. *Trichoplax* has sensory cells [42] and responds to food cues by altering its locomotion pattern [69].

Overall, the complete loss of a preexisting complex nervous system in two free-living lineages, the sponges and placozoans, both of which display active ciliary locomotion in at least one life cycle stage and possess sensory systems, appears improbable. If, however, the metazoan common ancestor had only a few protoneuron types, representing the precursors to advanced nervous systems, a scenario of simplification in sponges and placozoans and independent complexification in ctenophores and eumetazoans becomes tenable. Many cell types have been lost in evolution, including the follicle cells in leeches, colloblasts in *Beroe*, odontoblasts in birds and shell-secreting cells of nudibranchs. Similarly, a system of a few protoneuron types may have been lost or reduced in sponges and placozoans. Under such a scenario the large number of neuronal genes expressed in some *Trichoplax* cell types, including putative sensory-neurosecretory cells [19, 37], may represent the remnants of a proto-nervous system. Ctenophores and eumetazoans may then have evolved a complex nervous system independently, from the same precursor system, including protoneurons already able to communicate via a mixture of peptidergic, synaptic and electric signaling.

## Conclusions

We discussed available data on metazoan nervous systems in light of conflicting phylogenies. The phylogenetic position of the Ctenophora is still uncertain, and further analyses with better models, fuller taxon sampling or alternative markers will be needed to settle the question. We emphasized the methodological problems with the placement of ctenophores, arguing that the available data do not strongly challenge the traditional view of sponges occupying a position as a sister group of the remaining eumetazoans. This phylogeny is compatible with the single origin and stepwise evolution of nervous systems within the Neuralia [70]. If, however, future studies will provide better support for the ‘ctenophores first’ phylogeny, we favor the view of an independent complexification of nervous systems from a simple precursor state. Under this scenario the origin of nervous systems seems to be similar to the origin of complex visual eyes across Bilateria, with possibly very humble beginnings of homologous constituent photoreceptors and pigment cells evolving independently into complex eyes [71].

## Abbreviations

ASIC: Acid-sensing ion channel
DEG/ENaC: Degerin/ epithelial Na + channel
EST: expressed sequence tag
GABA: Gamma-aminobutyric acid
GPCR: G-protein-coupled receptor
LCA: Last common ancestor
LBA: Long branch attraction
LCA-LBA: Last common ancestor-long branch attraction.
